# The Persian Self-Report Version of the Antisocial Process Screening Device (APSD-P): A Psychometric Evaluation

**DOI:** 10.3389/fpsyt.2021.760531

**Published:** 2021-11-02

**Authors:** Ali Ebrahimi, Mojtaba Elhami Athar, Mona Darvishi, Olivier F. Colins

**Affiliations:** ^1^Student Research Committee, School of Behavioral Sciences and Mental Health (Tehran Institute of Psychiatry), Iran University of Medical Sciences, Tehran, Iran; ^2^Department of Clinical Psychology, School of Behavioral Sciences and Mental Health (Tehran Institute of Psychiatry), Iran University of Medical Sciences, Tehran, Iran; ^3^Department of Health Psychology, School of Behavioral Sciences and Mental Health (Tehran Institute of Psychiatry), Iran University of Medical Sciences, Tehran, Iran; ^4^Department of Special Needs Education, Faculty of Psychology and Educational Sciences, Ghent University, Gent, Belgium

**Keywords:** psychopathy, callous-unemotional traits, conduct problems, self-report, Persian version

## Abstract

The self-report version of the Antisocial Process Screening Device (APSD) is a commonly used tool for assessing psychopathic traits in youth. This is the first study designed to examine the factor structure, internal consistency, and convergent validity of the Persian APSD-SR in a sample of 675 school-attending youth in Iran (46% girls; M age = 16.35). Confirmatory factor analysis supported a modified three-factor model, with items loading on narcissism, callous-unemotional, and impulsivity dimensions, which was invariant across gender. Notwithstanding that the internal consistency of some APSD scores was unsatisfactory, the APSD total and dimension scores showed the expected relations with external correlates (e.g., conduct problems, aggression, and low prosocial behavior), supporting the validity of the interpretation of the APSD scores. The findings showed that the APSD is a useful tool for assessing psychopathic traits in Iranian adolescents and may spark research on adolescent psychopathy in mental health and forensic settings.

## Introduction

Psychopathic personality is a severe multifaceted personality disorder comprised of a constellation of co-occurring traits that load onto either three (1) or four (2) dimensions, including interpersonal, affective, behavior/lifestyle, and antisocial dimensions. Adults with a psychopathic personality have a disproportionate negative emotional and psychological impact on the lives, health, and careers of many others and incur tremendous costs and burdens to society (3–5). They also are reputed for resistance to treatment and confront clinicians with myriad challenges (6). To understand its etiology and improve intervention success, the multidimensional adult psychopathy construct has been given significant research attention in childhood and adolescence. Such research attention is highly relevant for numerous reasons. Adults with a psychopathic personality often commit crimes or excel in behaviors that are not technically illegal but negatively impact the well-being of others (7). Thus, early identification of children and adolescents at risk for adult psychopathic personality may help to protect society against future harm. Early detection of these individuals can also expand our understanding of the etiology and the development of psychopathy over time and the identification of protective factors hindering its development (8). Finally, the early detection of individuals with psychopathy may also generate knowledge that can be used to increase the effectiveness of prevention and intervention programs long before the early psychopathic behaviors become entrenched, chronic, and co-morbid with other problems (9). For all these reasons, the development of reliable tools for assessing psychopathic traits in children and adolescents is of utmost importance (10).

One of the most widely used tools for assessing psychopathic traits in youngsters is the Antisocial Process Screening Device [APSD, (11)]. The APSD was designed to assess as many of the Psychopathy Checklist-Revised [PCL–R; (12)] items as possible, though some items were excluded because they were not relevant for children (13, 14). Even before Cooke and Michie (1) favored a three-factor conceptualization of PCL–R measured psychopathy (which essentially excludes the antisocial dimension), the developer of the APSD favored a three-factor model in which the 18 of the 20 APSD items load on interpersonal (labeled Narcissism), affective (labeled Callous–Unemotional), and behavioral/lifestyle (labeled Impulsivity) dimensions (13). The sole APSD item that directly refers to criminal behavior (“*You engage in illegal activities”*) is only included in the total score, which implies that the APSD does not involve a fourth antisocial dimension. Originally, the APSD was designed as a parent and teacher rating tool to be used in 6–13-year-olds (14). Because of concerns that the validity of parent and teacher report decrease in adolescence, because parents and teachers are not always available, and because youth seems especially important when assessing features that may not be evident for observers, a self-report version of the APSD was developed later (11, 15).

Since the introduction of APSD (13), studies have evaluated the psychometric properties of the APSD-SR in forensic and community samples. While some studies supported a two-factor model of the APSD among students (14) and male and female adjudicated youths in the US (16), others have examined the three-factor model. For instance, Vitacco et al. (17) confirmed the proposed three-factor structure of the APSD-SR in a gender-mixed sample of detained youth, though the factor loadings of two items (items 19 and 20) did not reach the minimum threshold factor loading of 0.30. Several other studies have replicated the three-factor model of APSD-SR, but only after allowing some modifications. For example, Poythress et al. (18) examined the psychometrics of APSD-SR with female youths in a juvenile diversion program and reported a satisfactory fit for the three-factor model after removing items 19 and 20, a finding that was replicated by Douglas et al. (19). Similarly, Laajasalo et al. (20) replicated the three-factor model with the Finnish community male and female adolescent sample, though only after removing item 19, which did not load on any of the factors. A series of other studies, however, could not replicate the proposed three-factor structure of the APSD-SR, including studies in criminal justice-involved youth in Russia (21), detained female adolescents in Belgium (22), inmate delinquent and community youth in Portugal (23), and community-residing youth in China (24).

Across studies, the internal consistency of APSD-SR Total and factors scores, overall, was at least acceptable, with the exception of the Callous-Unemotional factor, whose internal consistency was often poor or unacceptable, both in the community (15, 23, 25) and forensic samples (18, 26). APSD-SR scores are generally related to psychopathy scores as measured by other self-report measures [e.g., Youth Psychopathic traits Inventory; (27)], supporting the convergent validity of the interpretation of APSD-SR scores (18, 22, 28). In support of their criterion validity, APSD scores showed expected associations with theoretically and clinically relevant features, such as conduct problems, aggression, peer problems, and prosocial behavior (13, 15, 22, 29–33).

### This Study

While APSD-SR is a widely used research measure in Western samples, it is unclear if the findings from Western samples are generalizable to Iran.

There are meaningful differences concerning interpersonal relationships, cultural values, and social norms (34), and emotional expression (35) between Eastern/Asian (e.g., Iran) and Western (e.g., Europe, USA) cultures (36, 37). In contrast to Western cultures, Eastern cultures encourage low arousal emotions (38). Therefore, restraining emotional expression might explain why Eastern/Asian children exhibit higher levels of callous-unemotional (CU) traits than children in the West (39–41). Also, lower levels of conduct problems have been observed in Eastern/Asian culture compared to Western countries (42), so it is possible that APSD-SR scores in Iran will not be as strongly related to conduct problems as in Western societies. Furthermore, Shariat et al. (43) found that in contrast to US samples, the superficial, deceitful, and grandiose items of the Psychopathy Checklist: Screening Version (PCL: SV) could not adequately differentiate the Iranian participants with psychopathy from their counterparts without psychopathy. This difference might be due to a cultural characteristic of the Iranian society, namely, “*ta'arof* ” - “the great national trait of exaggerated politesse, modesty, and self-deprecation that Iranians seem to be born with” [(44), p. 65]. Iran is a collectivistic society where people tend to conform to social expectations and prefer group harmony over personal desires and ambitions (43); thus, people may be deceitful in this context, but such conduct cannot be considered pathological since it does not deviate considerably from cultural expectations (43). Also, while being too superficial and charming may be related to problematic behaviors in North America, in Iran, those traits could not be regarded as pathological or impairment because of ta'arof, and may therefore not related to problematic behavior in Iranian citizens. Finally, there are marked cultural variations concerning the lack of empathy and remorse, and items related to these concepts demonstrated higher discriminatory power in Iran (43). Consequently, these traits could be greatly discriminant even at the lower levels. More specifically, collectivistic societies such as Iran may be more sensitive to self-centered emotions and affects, which could be recognized as a deviation. Thus, if a subject lacks remorse and empathy, he/she will be readily considered a psychopath by Iran's collectivistic people (43).

Taken together, unique cultural features may impact the expression of psychopathic traits in Iran, making it uncertain if findings from APSD-SR studies hold in Iran. Therefore, this present study was designed to examine the psychometric properties of the Persian APSD-SR in Iranian adolescents.

We examined the factor structure, the reliability, and the validity of APSD-SR in a sample of 700 Iranian school-attending adolescents. First, to test the proposed three-factor structure of the APSD-SR, confirmatory factor analyses (CFAs) will be performed. To enable comparison with prior work on the APSD-SR, we also tested if and expected the obtained factor structure to be invariant across gender. Second, to scrutinize the reliability of the Persian APSD-SR scores, reliability indices values will be calculated. Third, to test the convergent/ divergent validity of the APSD-SR scores, the current study includes variables that have been considered in prior APSD-SR studies. Specifically, it is hypothesized that APSD-SR factors and the total score would, overall, be positively related to conduct problems (45, 46), anger (47, 48), aggression (28, 47), peer problems (22, 23), and attention problems (22, 49) and negatively associated with prosocial behavior (50, 51). Since males typically exhibit higher mean levels of psychopathic traits and most of the external correlates (e.g., conduct problems) than females [e.g., (52)], the current study will examine if the pattern of the correlations between APSD scores and external correlates differs across gender.

## Materials and Methods

### Participants

Participants were 14–18 years old students from eight schools in Tehran who were recruited between November 2018 to April 2019. Specifically, four districts of Tehran city were selected randomly, and then eight schools from the selected districts were chosen. Finally, 25 classes (a total of ± 750 eligible students) from these eight schools were selected randomly, and the questionnaires were distributed to 700 students in the classes, and 675 participants (*M* age = 16.35; *SD* = 0.82; 46% girls) completed questionnaires (response rate: 96.4%). The gender groups were not matched with respect to age; girls (M: 16.47, *SD*: 0.80) had significantly higher mean age than (*t*_(675)_: 3.59, *p* < 0.01) boys (M: 16.25, *SD*: 0.82), though the magnitude of this difference was in the small range (Cohen's d = 0.28) (53).

### Procedure

The ethics committee of the Iran University of Medical Sciences first approved this study (code number: IR.IUMS.REC1395.95-04-193-29860). Next, approval was provided by the Iran Ministry of Education and boards of each school. For the present study, the original version of the Antisocial Process Screening Device Self-Report (APSD-SR) was translated to Persian by two translators who were fluent in English. Subsequently, Persian translations were compared and merged together and translated back from Persian to English by a third, independent translator. Next, the back-translated English version of the APSD-SR was made available for being administered after incorporating some revisions. Students, their parents, and teachers were informed about the survey administration. All students were surveyed unless they declined to participate or when their parents objected. The administration of the survey was conducted in the classroom on a regular school day. Before starting the assessment, the students were informed again about the confidentiality of the information and signed the consent form. They were asked to complete the questionnaires in their classroom during a 1-h session under the supervision of a specially trained research assistant (master-level student). Students could ask the supervisor for clarification if they did not understand the question. After the students finished their questionnaires, they brought them to the class box that was sealed by the research assistant.

### Measures

#### Antisocial Process Screening Device Self-Report (APSD-SR)

APSD-SR (11) includes 20 items that tap psychopathic traits and antisocial behavior in adolescents. Each item is graded on a three-point Likert scale, ranging from 0 (*no, not true in all cases*) to two (*certainly true*). Factor analyses (11) revealed a three-factor model for the APSD-SR, consisting of Narcissism (seven items), Callous-Unemotional (6 items), and Impulsivity (five items). Two items (items two and six) did not load onto any factor and are only used to calculate the total score [Frick and Hare, (11)]. The sum of these 20 items yields a total score for the APSD-SR.

#### The Aggression Questionnaire (AQ)

The Aggression Questionnaire (AQ) (54) has 29 items that need to be answered on a five-point Likert scale, ranging from one (i.e., *extremely uncharacteristic of me*) to five (i.e., *extremely characteristic of me*). AQ assesses four behavioral factors, including Physical Aggression (nine items; e.g., “*I get into fights a little more than the average person”*), Verbal Aggression (five items; e.g., “*I often find myself disagreeing with people”*), Anger (seven items; e.g., “*When frustrated, I let my irritation show”*), and Hostility (eight items; e.g. “*At times I feel I have gotten a raw deal out of life”*). These factors are categorized into three components, namely, a Motor or Instrumental component (physical and verbal aggression), an Emotional component (Anger), and a Cognitive component (Hostility). Mohammadi (55) supported the validity and reliability of the original four factors of the AQ when using the Persian AQ version. Cronbach's alpha and MICs for these factors can be retrieved from Table 1.

**Table 1:** Descriptive statistics of APSD-SR, AQ, and SDQ.

	**Total sample (*****n*** **= 675)**				**Boys (*****n*** **= 359)**				**Girls (*****n*** **= 316)**			
**Measures**	**Mean (*SD*)**	**α**	**MIC**	**ω**	**Mean (*SD*)**	**α**	**MIC**	**ω**	**Mean (*SD*)**	**α**	**MIC**	**ω**
APSD-SR _ total (17 items)	10.87 (4.62)	0.69	0.12	0.69	11.28 (4.66)	0.68	0.11	0.68	10.40 (4.53)	0.70	0.12	0.70
Narcissism (5 items)	2.89 (1.95)	0.54	0.19	0.55	3.10 (2.04)	0.54	0.19	0.55	2.66 (1.83)	0.52	0.18	0.55
Callous-Unemotional (5 items)	2.91 (1.94)	0.52	0.18	0.47	3.24 (1.88)	0.42	0.12	0.44	2.75 (1.80)	0.46	0.15	0.49
Impulsivity (5 items)	3.87 (1.88)	0.46	0.14	0.47	3.79 (1.92)	0.47	0.15	0.48	3.94 (1.85)	0.45	0.14	0.46
AQ _ total	83.32 (16.84)	0.84	0.15	0.84	83.54 (16.76)	0.83	0.15	0.84	82.95 (16.93)	0.83	0.14	0.84
Anger	20.36 (5.67)	0.68	0.22	0.69	19.63 (5.61)	0.66	0.21	0.68	21.15 (5.63)	0.67	0.23	0.69
Hostility	23.51 (5.82)	0.65	0.19	0.66	23.42 (5.63)	0.62	0.17	0.62	23.63 (6.06)	0.68	0.21	0.68
Aggression (Physical)	24.76 (6.41)	0.63	0.16	0.67	25.94 (6.07)	0.59	0.14	0.64	23.34 (6.45)	0.66	0.18	0.69
Aggression (Verbal)	14.68 (3.45)	0.42	0.15	0.33	14.53 (3.50)	0.36	0.10	0.37	14.82 (3.39)	0.28	0.07	0.28
SDQ _ total	21.70 (5.60)	0.63	0.06	0.54	21.22 (5.43)	0.60	0.06	0.47	25.21 (4.96)	0.66	0.07	0.65
Emotional problems	3.78 (2.36)	0.65	0.27	0.65	3.40 (2.10)	0.56	0.20	0.57	4.24 (2.55)	0.70	0.32	0.70
Conduct problems	3.08 (2.00)	0.50	0.17	0.52	3.27 (1.97)	0.48	0.15	0.49	2.86 (1.83)	0.55	0.20	0.58
Hyperactivity problems	4.15 (2.08)	0.51	0.17	0.51	3.93 (1.98)	0.46	0.14	0.46	5.57 (1.68)	0.56	0.20	0.56
Peer problems	3.51 (2.09)	0.45	0.15	0.47	3.83 (1.99	0.40	0.12	0.41	4.90 (1.56)	0.48	0.16	0.49
Prosocial behavior	7.18 (2.12)	0.65	0.28	0.65	6.82 (2.17)	0.64	0.26	0.64	7.60 (1.99)	0.65	0.27	0.65

*APSD-SR, Antisocial Process Screening Device - Self-Report; AQ, Aggression Questionnaire; SDQ, Strengths and Difficulties Questionnaire; α, Chrobach's Alpha; MIC, mean interitem correlation; ω, McDonald's Omega*.

#### The Strengths and Difficulties Questionnaire: Self-Report Version (SDQ)

The self-report version of the SDQ includes 25 items and assesses the psychosocial adjustment of children and adolescents (56). The SDQ includes five subscales, being Conduct Problems (e.g., “*I take things that are not mine from home, school or elsewhere”*), Emotion Problems (e.g., “*I get a lot of headaches, stomach-aches or sickness”*), Peer Problems (e.g., “*I have one good friend or more”*), Prosocial (e.g., “*I am kind to younger children”*), and Hyperactivity (e.g., “*I am constantly fidgeting or squirming”*). Each subscale consisting of 5 items with three response categories (*not true* = 0, *somewhat true* = 1, *certainly true* = 2) (56, 57). SDQ is currently available in various languages, including Persian. A higher score means that the adolescent experiences more difficulties, with the exception of a higher prosocial behavior score, which indicates less problems. Tehrani Doust et al. (58) examined the psychometrics of the Persian version of the SDQ and supported the reliability and validity of the Persian SDQ scores. Cronbach's alpha and MICs for the five SDQ subscales can be retrieved from Table 1.

### Data Analysis

APSD-SR data (ranging from 1–20 items) were missing for 27 participants. To include as many cases as possible, missing values were handled using the series mean method in SPSS 18.0; also, the Boxplot method was used to address outliers, resulting in a sample size of 675. Descriptive statistics were calculated for study variables and are presented in Table 1 In order to test the proposed two-factor (14) and three-factor (11) structure of the APSD-SR, we conducted confirmatory factor analyses (CFAs) with Diagonally Weighted Least Squares (DWLS) using the JASP free software. The DWLS (WLSMV or robust WLS in *Mplus* software) yields less biased and more accurate results than other procedures in every condition, especially with ordinal data (59). Model fit was assessed using the Tucker–Lewis index (TLI), the comparative fit index (CFI), and the root mean square error of approximation (RMSEA). We considered RMSEA scores below 0.05 to indicate a good fit and scores between 0.05 and 0.08 indicating acceptable fit. A TLI and CFI score of 0.95 or above indicates excellent fit, and scores of 0.90 or more indicate a good fit (60, 61). The three-factor model was specified with the 18 items as observed variables and the three factors as latent and correlated constructs. In line with prior work that tested a two-factor model (22, 23), this model was specified with 16 items and two latent factors, being impulsivity/conduct problems (10 items) and callous-unemotional (six items). Also, using the best fitting model we performed measurement invariance (MI) tests across gender groups based on the sequential strategy suggested by Meredith and Teresi (62). Since the model should initially fit both groups, the selected model was tested separately for boys and girls as a first step of the procedure. Three levels of MI (i.e., configural, metric, and scalar) were tested to examine whether the factor structure, factor loadings, and item intercepts, respectively, were invariant across groups. Change in CFI (ΔCFI) was used as an indicator for testing MI which is independent of model parameters and sample size. According to Cheung and Rensvold (63), a value of CFI smaller than or equal to 0.01 supports the presence of MI across groups.

The internal consistency of the APSD-SR scores was examined using Cronbach's alpha (α), mean inter-item correlation (MIC), and Macdonald's Omega (ω) values. Cronbach's alpha reliability coefficient ranges between 0 and 1, and the closer it is to 1.0, the greater the internal consistency of the items in the scale. George and Mallery (64) provide the following rules of thumb for Cronbach's alpha coefficient: “ >0.9 = Excellent; >0.8 = Good; >0.7 = Acceptable; >0.6 = Questionable; >0.5 = Poor; and 0.5 > = Unacceptable” (p. 231). In contrast to α, MIC values are not dependent on the number of items in a scale and should be in the range of 0.15–0.50 to be considered adequate (65). Also, a threshold for Macdonald's ω > 0.70 was considered satisfactory according to Nunnally and Bernstein criterion (66). The item-total and item-factor correlations were also performed for APSD-SR scores, which are presented in Table 2.

**Table 2:** Item-total and item-factor correlations of APSD-SR (*n* = 675).

	**APSD_Total**	**NAR**	**CU**	**IMP**
APSD_total	1			
Nar	0.79^**^	1		
CU	0.65^**^	0.30^**^	1	
IMP	0.69^**^	0.40^**^	0.16^**^	1
Item 1	0.34^**^	0.26^**^	0.05	0.47^**^
Item 2	0.44^**^	0.26^**^	0.46^**^	0.26^**^
Item 3 (R)	0.47^**^	0.19^**^	0.66^**^	0.18^**^
Item 4	0.41^**^	0.26^**^	0.12^**^	0.54^**^
Item 5	0.45^**^	0.55^**^	0.14^**^	0.28^**^
Item 6	0.50^**^	0.41^**^	0.14^**^	0.28^**^
Item 7 (R)	0.40^**^	0.21^**^	0.52^**^	0.16^**^
Item 8	0.45^**^	0.59^**^	0.18^**^	0.19^**^
Item 9	0.28^**^	0.14^**^	−0.07	0.59^**^
Item 10	0.61^**^	0.68^**^	0.28^**^	0.32^**^
Item 11	0.51^**^	0.59^**^	0.22^**^	0.30^**^
Item 12 (R)	0.31^**^	0.14^**^	0.61^**^	−0.05
Item 13	0.43^**^	0.21^**^	0.18^**^	0.57^**^
Item 14	−0.12^**^	0.01	−0.25^**^	−0.02
Item 15	0.02	0.10^**^	−0.18^**^	0.13^*^
Item 16	0.33^**^	0.54^**^	0.08^*^	0.10^*^
Item 17	0.48^**^	0.26^**^	0.17^**^	0.62^**^
Item 18 (R)	0.22^**^	0.05	0.53^**^	−0.06
Item 19	0.04	0.07	−0.07	0.06
Item 20 (R)	0.33^**^	0.17^**^	0.29^**^	0.01

*NAR, Narcissism; IMP, Impulsivity; CU, Callous-Unemotional; R, reversed;*

** *p < 0.001;*

* *p < 0.05*.

Finally, to evaluate the convergent validity of the interpretation of the APSD-SR factor and total scores, Pearson correlation coefficients were examined between the ASPD-SR scores and external correlates of interest (e.g., conduct problems, aggression, and prosocial behavior). Hemphill (67) suggested that the coefficients ≤ 0.10 are indicative of weak; 0.20–0.29 suggest moderate, and ≥0.30 indicate strong correlations. An alpha of *p* < 0.05 was used as an indicator for statistical significance. All analyses were performed using SPSS 20 unless otherwise specified.

## Results

### Confirmatory Factor Analysis

**Figure 1:**
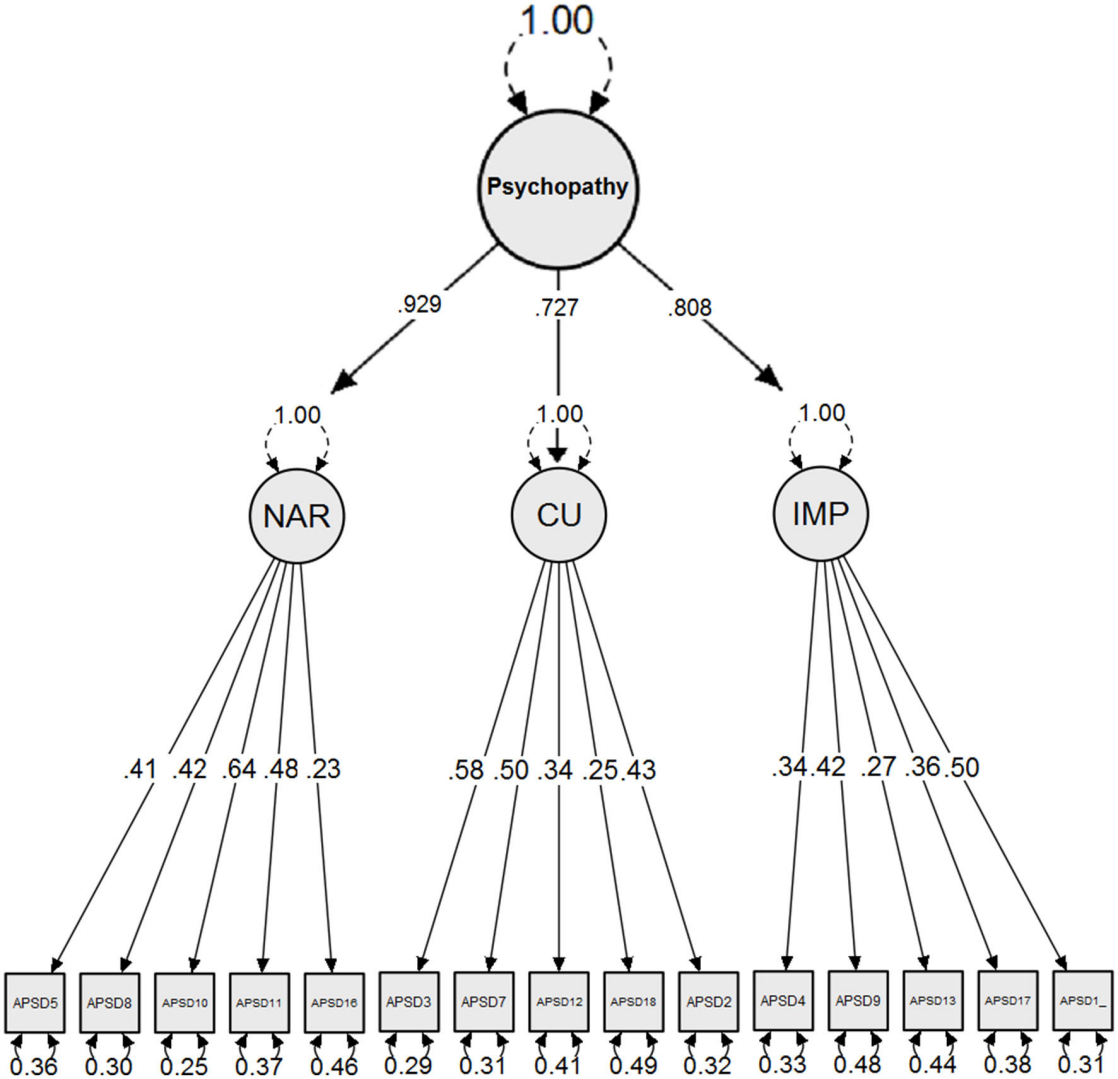
Confirmatory factor analysis model (*n* = 675). NAR, Narcissism; CU, Callous-Unemotional; IMP, Impulsivity.

Neither the two-factor (RMSEA = 0.060; CFI = 0.77, TLI = 0.71) nor the proposed three-factor models (RMSEA = 0.059; CFI = 0.78, TLI = 0.75) reached adequate fit. Table 3 shows that items 15 and 19 did not significantly load on the Narcissism and Callous-Unemotional factor, respectively, whereas item 14 loaded negatively on Narcissism. When removing these three items from the CFA, the three-factor model reached adequate fit (RMSEA = 0.038; CFI = 0.92; TLI = 0.91) (see Figure 1). The two-factor and three-factor models of the APSD-SR were also tested for boys and girls separately. While the two-factor model did not reach adequate model fit for boys (RMSEA = 0.052; CFI = 0.80, TLI = 0.77) and girls (RMSEA = 0.053; CFI = 0.81, TLI = 0.78), the three-factor model yielded an acceptable model fit for boys (RMSEA = 0.034; CFI = 0.94, TLI = 0.92) and girls (RMSEA = 0.034; CFI = 0.94, TLI = 0.93), but only after removing items 14, 15, and 19. Then, configural, metric, and scalar invariance were examined in sequence for gender groups. Model fit indices was in the acceptable range for configural (RMSEA = 0.034, CFI = 0.94, TLI = 0.93), metric (RMSEA = 0.037; CFI = 0.92; TLI = 0.91) and scalar invariance (RMSEA = 0.040, CFI = 0.90, TLI = 0.91). These results indicate that the modified three-factor model of the APSD-SR was invariant across gender groups [ΔCFIs ≤ 0.01; (63)]. Therefore, all results reported below were based on this modified three-factor model, which also implies that items 14, 15, and 19 are not used to calculate the APSD Total score. To enhance comparison with APSD-SR based research in Western societies, all analyses reported from here onwards were repeated whilst including these three items in the factors and total scores. Results from these analyses can be retrieved from the Supplementary Material but will neither be referred to in the Result section nor reflected upon in the Discussion.

**Table 3:** APSD-SR item loadings.

**Item**	**NAR**	**CU**	**IMP**
5. Your emotions are shallow and fake.	0.41^*^		
8. You brag a lot about your abilities, accomplishments, or possession.	0.41^*^		
10. You use or “con” other people to get what you want.	0.62^*^		
11. You tease or make fun of other people.	0.47^*^		
14. You can act charming and nice to get what you want.	−0.14^*^		
15. You get angry when corrected or punished.	0.04		
16. You think you are better or more important than other people.	0.22^*^		
3. You care about how well you do at school or work. (R)		0.58^*^	
7. You are good at keeping promises. (R)		0.50^*^	
12. You feel bad or guilty when you do something wrong. (R)		0.34^*^	
18. You are concerned about the feelings of others. (R)		0.25^*^	
19. You hide your feelings or emotions from others.		−0.01	
20. You keep the same friends. (R)		0.43^*^	
1. You blame others for your mistakes.			0.34^*^
4. You act without thinking of the consequences.			0.42^*^
9. You get bored easily.			0.27^*^
13. You do risky or dangerous things.			0.34^*^
17. You do not plan ahead or leave things until the “last minute.”			0.51^*^

*NAR, Narcissism; IMP, Impulsivity; CU, Callous-Unemotional; R, reversed;*

* *T-value (p < .05)*.

### Internal Consistency and Correlations Between the APSD-SR Scores

As shown in Table 1, the internal consistency of the modified APSD-SR factor scores expressed by α for the total sample and boys sample were in the unacceptable to questionable ranges, and for the girls sample ranged from unacceptable (Impulsivity = 0.45) to acceptable range (Total Score = 0.70). When relying on the MIC as an index of internal consistency, for the total sample, the Narcissism and Callous-Unemotional factor scores were indicative of acceptable internal consistency, though the APSD-SR total score and Impulsivity factor score had inadequate internal consistency (Table 1). In the same vein, only the Narcissism and Impulsivity factor scores yielded adequate MIC values in the boys sample, whereas only the Narcissism and Callous-Unemotional factor scores demonstrated acceptable MIC values in the girls sample. With respect to Macdonald's ω, APSD-SR Total and factor scores did not demonstrate internal consistency in the total and boys sample, and only the APSD-SR Total score in the girls sample reached the threshold of 0.70. Significant zero-order correlations were also found between APSD-SR factor scores and the APSD-SR total score and between the three APSD-SR factor scores, and the patter of correlations was consistent across gender. These correlations were: r^CU−total^ = 0.65; r^IMP−total^ = 0.69; r^NAR−total^ = 0.79; r^CU−NAR^ = 0.30; r^NAR−IMP^ = 0.40; r^CU−IMP^ = 0.16.

### Convergent/Divergent Validity

As shown in Table 3, the APSD-SR total score was positively related to anger, physical and verbal aggression, hostility, emotional problems, hyperactivity problems, peer problems, and conduct problems, but negatively with prosocial behavior. All three APSD-SR factor scores were negatively associated with prosocial behavior but positively with anger, physical and verbal aggression, conduct problems, hyperactivity problems, and peer problems. In addition, the Narcissism and Impulsivity factor scores, but not the Callous-Unemotional factor score, were significantly and positively associated with emotional problems and hostility (Table 3). Altogether, the pattern of correlations was consistent across genders, with one notable exception: the association between Narcissism and emotional problems was only significant in the boys sample, not in the girls sample (Table 4).

**Table 4:** Correlations between APSD-SR scores and between APSD-SR scores and external correlates.

	**Total sample (*****n*** **= 675)**				**Boys (*****n*** **= 359)**				**Girls (*****n*** **= 316)**			
**Measures**	**APSD total**	**Narcissism**	**Callous – unemotional**	**Impulsivity**	**APSD total**	**Narcissism**	**Callous – unemotional**	**Impulsivity**	**APSD total**	**Narcissism**	**Callous – unemotional**	**Impulsivity**
APSD _ total	1	–	–	–	1	–	–	–	1	–	–	–
Narcissism	0.79^**^	1	–	–	0.79^**^	1	–	–	0.79^**^	1	–	–
Callou Unemotional	0.65^**^	0.30^**^	1	–	0.63^**^	0.26^**^	1	–	0.68^**^	0.34^**^	1	–
Impulsivity	0.69^**^	0.40^**^	0.16^**^	1	0.70^**^	0.41^**^	0.14^**^	1	0.69^**^	0.40^**^	0.21^**^	1
AQ_total	0.45^**^	0.36^**^	0.18^**^	0.41^**^	0.39^**^	0.33^**^	0.12^*^	0.35^**^	0.52^**^	0.41^**^	0.26^**^	0.48^**^
Anger	0.36^**^	0.27^**^	0.12^**^	0.38^**^	0.37^**^	0.30^**^	0.12^*^	0.35^**^	0.39^**^	0.27^**^	0.16^**^	0.41^**^
Hostility	0.31^**^	0.29^**^	0.04	0.32^**^	0.26^**^	0.25^**^	0.02	0.26^**^	0.36^**^	0.39^**^	0.07	0.39^**^
Aggression (Physical)	0.43^**^	0.34^**^	0.27^**^	0.32^**^	0.35^**^	0.29^**^	0.11^*^	0.32^**^	0.51^**^	0.34^**^	0.39^**^	0.37^**^
Aggression (Verbal)	0.27^**^	0.21^**^	0.13^**^	0.23^**^	0.23^**^	0.18^**^	0.12^*^	0.16^**^	0.33^**^	0.27^**^	0.15^**^	0.32^**^
SDQ_total	0.28^**^	0.20^**^	0.02	0.37^**^	0.26^**^	0.23^**^	0.01	0.30^**^	0.33^**^	0.21^**^	0.04	0.46^**^
Emotional problems	0.18^**^	0.14^**^	−0.03	0.27^**^	0.26^**^	0.23^**^	0.04	0.25^**^	0.15^**^	0.10	−0.06	0.29^**^
Conduct problems	0.42^**^	0.30^**^	0.29^**^	0.29^**^	0.33^**^	0.27^**^	0.20^**^	0.21^**^	0.52^**^	0.33^**^	0.36^**^	0.40^**^
Hyperactivity problems	0.33^**^	0.19^**^	0.15^**^	0.37^**^	0.28^**^	0.20^**^	0.11^*^	0.27^**^	0.41^**^	0.22^**^	0.22^**^	0.48^**^
Peer problems	0.21^**^	0.19^**^	0.11^**^	0.14^**^	0.19^**^	0.19^**^	0.05	0.12^*^	0.19^**^	0.16^**^	0.01	0.18^**^
Prosocial behavior	−0.36^**^	−0.27^**^	−0.39^**^	−0.09^*^	−0.31^**^	−0.25^**^	−0.33^**^	−0.06	−0.40^**^	−0.27^**^	−0.42^**^	−0.15^**^

*APSD-SR, Antisocial Process Screening Device - Self-Report; AQ, Aggression Questionnaire; SDQ, Strengths and Difficulties Questionnaire;*

** *p < 0.001;*

* *p <0.05*.

## Discussion

This study aimed to examine the psychometric properties and factor structure of APSD-SR among a sample of Iranian school attending adolescents. The first aim was to test the proposed three-factor structure of the APSD-SR. Results did not yield an adequate fit for this model (or for the two-factor model). After excluding three items (14, 15, and 19), findings from CFA supported the three-factor model of APSD-SR, which was also invariant across gender groups. This suggests that this modified APSD-SR assessment tool measures three distinct but interrelated dimensions (or factors or components) of the psychopathy construct. Item 14 (“*You act charming and nice to get things you want”*), which was removed due to its negative loading on the Narcissism factor, and had a lower factor loading in several other studies too, suggesting that our finding is consistent with the literature (17, 21, 24). We also excluded item 15 (“*You get angry when corrected or punished*”) from the Narcissism factor to reach an acceptable fit. Prior work also showed that this item was not a good indicator of the Narcissism scale. For example, in studies with Chinese, Spanish, and Dutch samples, item 15 loaded on the Impulsivity factor (24, 28, 68). Finally, item 19 (“*you hide your feelings and emotions from others*”) was also eliminated because of having a non-significant loading on the Callous-unemotional factor, a finding that again dovetails with prior work (18, 20). Taken together, our findings and their consistency with past research suggest that the modification indices are not sample specific and even generalizes to non-Western cultures.

Conceptually, the APSD component scores should measure interrelated aspects of the same overarching construct of psychopathic personality. Therefore, it would follow that these factors should demonstrate moderate to strong associations with one another. However, this study showed that the correlations between these APSD factor scores were in the moderate range at best (Narcissism-Impulsivity = 0.40; Narcissism-Callous-Unemotional = 0.30; Callous-Unemotional-Impulsivity = 0.16. *p* < 0.001). Altogether, these correlation coefficients are relatively low for dimensions that are part of the same construct, especially the relation between the Callous-Unemotional and Impulsivity scores. In addition, based on adult literature (2), Narcissism and Callous-Unemotional components are expected to be more strongly related than the Narcissism and Impulsivity components and the Callous-Unemotional and Impulsivity components (69). However, the current study showed that the APSD Narcissism score was stronger related to the APSD Impulsivity than to the APSD Callous-Unemotional component score, a finding that is in line with previous APSD-SR studies (22, 70).

The results showed that when using Cronbach's alpha (α), the internal consistency of the APSD-SR scores ranged from unacceptable (Impulsivity) over poor (Narcissism and Callous-Unemotional) to questionable (Total scores in the total and boys sample) and acceptable (Total score in girls sample). However, if α penalizes shorter scales, then it should not come as a surprise to find low α estimates for scales with relatively few items, such as the APSD component scores. Yet, when using the mean interitem correlation (MIC) as an index for internal consistency that is not affected by the number of items in a scale, for the total sample, the internal consistency for the APSD Total and Impulsivity score was unacceptable, whilst the MIC estimates for the APSD Narcissism and Callous-Unemotional scores were only slightly above the threshold to be considered acceptable. Similarly, only Narcissism and Impulsivity scores had adequate MIC values in the boys sample, and only Narcissism and Callous-Unemotional scores yielded acceptable MIC values in the girls sample. However, concerning Macdonald's ω, only the APSD-SR Total score in the girls sample yielded adequate internal consistency. Possibly, the low internal consistency of the APSD-SR scores might be related to the tendency of respondents to answer questions in a manner that is viewed favorably by others (i.e., social desirability), which is reinforced by Iranian collectivistic culture. Notwithstanding, our results regarding the internal consistency of the APSD scores is consistent with prior work (15, 22, 24, 25). For instance, in the study of Colins et al. (22) only the APSD Total score had acceptable internal consistency, while all APSD subscales failed to reach acceptable alpha ranges. Similarly, in the Chinese version of the APSD (24), Chronbach's alpha coefficients were not in the acceptable range for the APSD Total score and its three subscales, while the three subscales reached acceptable reliability based on the MIC values. Prior work showed that higher α and MIC estimates were reported for other self-report rating scales with a similar number of items to tap the three psychopathy components, such as the Youth Psychopathic traits Inventory-Short Version (71, 72). Hence, it is possible that the difficulties in reaching at least acceptable internal consistency are tool-specific (APSD-SR). Yet, it remains to be seen how well-other self-report scales perform in Iranian samples.

The current study also examined correlations between APSD scores and external criterion measures to bolster what is known about the convergent/divergent validity of the Persian version of this self-report tool. Echoing prior work (13, 15, 22, 29–33), the APSD Total score was positively and significantly related to anger, aggression (verbal and physical), hostility, conduct problems, peer problems, and hyperactivity problems and negatively and significantly related to prosocial behavior. In the same vein, all three APSD-SR component scores were significantly negatively associated with prosocial behavior, but significantly positively associated with anger, physical and verbal aggression, conduct problems, hyperactivity problems, and peer problems. In addition, only Narcissism and Impulsivity were subscales that had significant positive associations with emotional problems and hostility. Also, correlations between the measures were performed separately for boys and girls, with the results indicating that the pattern of correlations was consistent across gender, except for the association between Impulsivity and peer problems, which was significant only in the girls sample but not in boys sample. Overall, the APSD component scores yielded the same pattern of correlations with external correlates that have were found in prior studies. In sum, our results provide support for the convergent/divergent validity of the interpretation of the modified APSD-SR total and component scores in Iranian school-attending adolescents.

All in all, despite its poor reliability, previous studies (17, 20, 25) and the current study showed that the proposed three-factor structure can be confirmed with some modifications and that these modified APSD scores were related to external correlates of interests in the hypothesized way.

As always, our findings should be interpreted in the context of some notable exaptation. First, we entirely relied on self-report information to measure convergent validity. Therefore, correlations between self-report measured psychopathy and external correlates may partly be explained by shared method variance. Second, the gender groups were not matched with respect to age, which implies that we cannot totally exclude the possibility that age differences have affected the results. Third, the cross-sectional nature of the current study does not allow conclusions about causality (e.g., between psychopathic traits and conduct problems) and prognosis (e.g., psychopathic traits as a predictor of future aggression). Third, participants were school-attending adolescents, and it cannot be excluded that adolescents who exhibit the highest levels of antisocial behavior have been absent the day the survey took place or did not want to participate. Future research must ascertain if findings can be generalized to Iranian samples of criminal justice-involved or conduct disordered youth.

## Data Availability Statement

The raw data supporting the conclusions of this article will be made available by the authors, without undue reservation.

## Ethics Statement

The studies involving human participants were reviewed and approved by Ethics Committee of the Iran University of Medical Sciences. Written informed consent to participate in this study was provided by the participants' legal guardian/next of kin.

## Author Contributions

AE: gathered data and prepared the manuscript. MEA: performed the data analysis and prepared the manuscript. MD: gathered data. OC: reviewed and revised the manuscript. All authors contributed to the article and approved the submitted version.

## Funding

This study was financially supported by the Student Research Committee, Iran University of Medical Sciences, Tehran, Iran (grant number 950419329707).

## Conflict of Interest

The authors declare that the research was conducted in the absence of any commercial or financial relationships that could be construed as a potential conflict of interest.

## Publisher's Note

All claims expressed in this article are solely those of the authors and do not necessarily represent those of their affiliated organizations, or those of the publisher, the editors and the reviewers. Any product that may be evaluated in this article, or claim that may be made by its manufacturer, is not guaranteed or endorsed by the publisher.
