# Supplementary material for: The Persian Self-Report Version of the Antisocial Process Screening Device (APSD-P): A Psychometric Evaluation
Source: Front Psychiatry. 2021 Nov 2;12:760531. doi: 10.3389/fpsyt.2021.760531 (PMC8594756; doi:10.3389/fpsyt.2021.760531)
Supplement: Supplementary file 1 [file Data_Sheet_1.pdf]

Supplementary material

Supplementary Table 1

Descriptive Statistics for the APSD-SR items (n = 675).

Item	*Min*	*Max*	*M*	*SD*	*SK*	*KU*
Item 1 - You blame others for your mistakes.	0	2	.57	.58	.46	-.65
Item 2 - You engage in illegal activities.	0	2	.50	.62	.83	-.30
Item 3 - You care about how well you do at school/work.	0	2	.50	.66	.97	-.20
Item 4 - You act without thinking of the consequences.	0	2	.66	.63	.41	-.64
Item 5 - Your emotions we shallow and fake.	0	2	.54	.66	.83	-.41
Item 6 - You lie easily and skillfully.	0	2	.68	.68	.50	-.80
Item 7 - You are good at keeping promises.	0	2	.57	.63	.66	-.53
Item 8 - You brag a lot about your abilities, accomplishments, or possessions.	0	2	.36	.60	1.42	.95
Item 9 - You get bored easily.	0	2	1.16	.71	-.25	-1.03
Item 10 - You use or "con" other people to get what you want.	0	2	.49	.65	.97	-.19
Item 11 - You tease or make fun of other people.	0	2	.69	.68	.48	-.83
Item 12 - You feel bad or guilty when you do something wrong.	0	2	.53	.67	.90	-.38
Item 13 - You do risky or dangerous things.	0	2	.82	.71	.26	-1.00
Item 14 - You act charming and nice to get things you want.	0	2	1.36	.63	-.47	-.65
Item 15 - You get angry when corrected or punished.	0	2	1.28	.68	-.44	-.84
Item 16 - You think you are better or more important than other people.	0	2	.79	.69	.31	-.93
Item 17 - You do not plan ahead or you leave things until the "last minute."	0	2	.63	.71	.67	-.78
Item 18 - You are concerned about the feelings of others.	0	2	.89	.72	.15	-1.06
Item 19 - You hide your feelings or emotions from others.	0	2	.92	.69	.09	-.92
Item 20 - You keep the same friends.	0	2	.40	.62	1.27	.49

Note. *APSD-SR*: Antisocial Process Screening Device-Self –Report; *Min*: Minimum; *Max*: Maximum; *M*: Mean; *SD*: Standard deviation; *SK*: Skewness: *KU*: Kurtosis

Supplementary Table 2

Descriptive Statistics of APSD-SR Original Version (n = 675).

Measures	Mean	Range	*SD*	Skewness	Kurtosis	*α*	MIC
APSD-SR _ Total (20 items)	14.44	3-30	4.75	.32	-.23	.64	.08
Narcissism (7 items)	5.54	0-14	2.26	.28	.17	.47	.11
Callous-Unemotional (6 items)	3.84	0-11	2.01	.61	.39	.40	.10
Impulsivity (5 items)	3.86	0-10	1.89	.19	-.17	.46	.14

Note. *APSD-SR*: Antisocial Process Screening Device-Self -Report; *AQ* = the Aggression Questionnaire; *SDQ* = Strengths and Difficulties Questionnaire; *SD* = Standard deviation; *α*: Chrobach's Alpha; *MIC* = mean interitem correlation

Supplementary Table 3

Pearson correlation of APSD-SR ,AQ, and SDQ (n = 675).

Measures	APSD Total	Narcissism	Callous – Unemotional	Impulsivity
APSD _ Total	1	-	-	-
Narcissism	.76**	1	-	-
Callous – Unemotional	.69**	.38**	1	-
Impulsivity	.54**	.10*	.09*	1
AQ_total	.47**	.36**	.17**	.41**
Anger	.39**	.29**	.12**	.38**
Hostility	.33**	.28**	.07	.32**
Aggression (Physical)	.44**	.34**	.21**	.32**
Aggression (Verbal)	.28**	.19**	.14**	.23**
SDQ_total	.31**	.21**	.05	.37**
Emotional problems	.20**	.14**	.03	.27**
Conduct problems	.43**	.29**	.25**	.29**
Hyperactivity problems	.12**	.17**	-.08**	.15**
Peer problems	.21**	.15**	.14**	.14**
Prosocial behavior	-.34**	-.19**	-.39**	-.09*

Note. *APSD-SR*: Antisocial Process Screening Device - Self-Report; *AQ*: Aggression Questionnaire; *SDQ*: Strengths and Difficulties Questionnaire $^{**}p < .001$; $^{*}p < .05$
